# (*R*)-2-Benzyl-4-methyl­pentyl (*R*)-2-meth­oxy-2-(1-naphth­yl)propionate

**DOI:** 10.1107/S160053681002101X

**Published:** 2010-06-16

**Authors:** Yoshinori Inoue, Takatoshi Matsumoto, Masataka Watanabe, Hiroshi Katagiri, Tsutomu Kumagai

**Affiliations:** a Department of Material Science, School of Engineering, The University of Shiga Prefecture, 2500 Hassaka-cho, Hikone, Shiga 522-8533, Japan; b Institute of Multidisciplinary Research for Advanced Materials, Tohoku University, 2-2-1 Katahira, Aoba, Sendai 980-8577, Japan; c Department of Chemistry and Chemical Engineering, Graduate School of Science and Engineering, Yamagata University, 4-3-16 Jonan, Yonezawa, Yamagata 992-8510, Japan

## Abstract

The relative configuration of the alcohol component in the title ester, C_27_H_32_O_3_, has been assigned as (*R*) from the known configuration of (*R*)-(−)-2-meth­oxy-2-(1-naphth­yl)propionic acid [(*R*)-MαNP acid]. In the crystal structure, the C atom of the methyl group of the MαNP acid lies in the extended plane of the naphthyl ring system [methyl C atom deviates from plane by 0.211 (2) Å; r.m.s. deviation of fitted atoms = 0.0187 Å] and a weak intra­molecular C—H⋯O hydrogen bond links the naphthyl ring system and the meth­oxy group. These structural properties are similar to those of most MαNP acid esters.

## Related literature

For general background to the crystalline-state analysis of 2-meth­oxy-2-(1-naphth­yl)propionic acid esters, see: Kuwahara *et al.* (2007 ▶); Sekiguchi *et al.* (2008 ▶). 
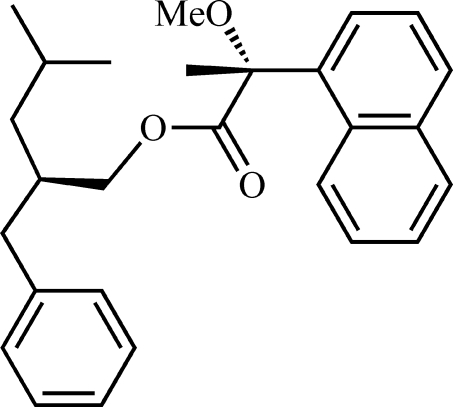


## Experimental

### 

#### Crystal data

C_27_H_32_O_3_
*M* *_r_* = 404.53
Monoclinic, 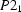

*a* = 9.3380 (1) Å
*b* = 12.4142 (1) Å
*c* = 10.0317 (5) Å
β = 102.8144 (8)°
*V* = 1133.95 (6) Å^3^
*Z* = 2
Cu *K*α radiation
μ = 0.59 mm^−1^
*T* = 105 K
0.60 × 0.60 × 0.60 mm

#### Data collection

Rigaku R-AXIS RAPID CCD diffractometer
Absorption correction: multi-scan (*ABSCOR*; Higashi, 1995 ▶) *T*_min_ = 0.896, *T*_max_ = 1.000
21388 measured reflections
4133 independent reflections
4042 reflections with *I* > 2σ(*I*)
*R*_int_ = 0.026

#### Refinement

*R*[*F* ^2^ > 2σ(*F* ^2^)] = 0.025
*wR*(*F* ^2^) = 0.068
*S* = 1.06
4133 reflections
276 parameters
1 restraint
H-atom parameters constrained
Δρ_max_ = 0.16 e Å^−3^
Δρ_min_ = −0.11 e Å^−3^
Absolute structure: Flack (1983 ▶), 1592 Friedel pairs
Flack parameter: 0.03 (13)

### 

Data collection: *PROCESS-AUTO* (Rigaku, 1998 ▶); cell refinement: *PROCESS-AUTO*; data reduction: *CrystalStructure* (Rigaku/MSC, 2003 ▶); program(s) used to solve structure: *SHELXS97* (Sheldrick, 2008 ▶); program(s) used to refine structure: *SHELXL97* (Sheldrick, 2008 ▶); molecular graphics: *PLATON* (Spek, 2009 ▶); software used to prepare material for publication: *Yadokari-XG 2009* (Wakita, 2001 ▶; Kabuto *et al.* (2009 ▶).

## Supplementary Material

Crystal structure: contains datablocks I, global. DOI: 10.1107/S160053681002101X/zs2041sup1.cif

Structure factors: contains datablocks I. DOI: 10.1107/S160053681002101X/zs2041Isup2.hkl

Additional supplementary materials:  crystallographic information; 3D view; checkCIF report

## Figures and Tables

**Table 1:** Hydrogen-bond geometry (Å, °)

*D*—H⋯*A*	*D*—H	H⋯*A*	*D*⋯*A*	*D*—H⋯*A*
C13—H13⋯O1	0.95	2.40	2.9887 (15)	120

**Fig. 1.**
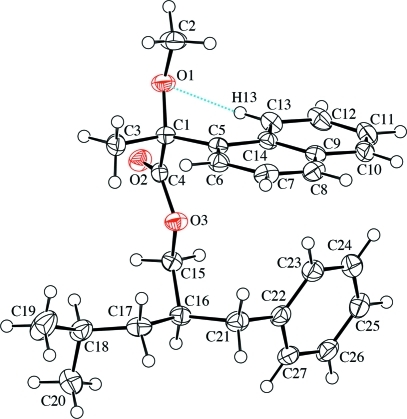
Molecular configuration and atom numbering scheme for the title compound, with displacement ellipsoids drawn at the 50% probability level.

## supplementary crystallographic information

### Comment

In a previous paper, we reported that (*S*)-2-methoxy-2-(1-naphthyl)propionic acid [(*S*)-MαNP acid] is an efficient auxiliary for enantioresolution of racemic secondary alcohols and the simultaneous determination of the absolute configuration of the resolved alcohols by the Advanced Mosher Method (Kuwahara *et al.*, 2007). We also reported the determination of the absolute configuration of esters condensed with (*S*)-MαNP acid using X-ray crystallography, by comparison with the known configuration of the asymmetric quaternary carbon of the acid as an internal standard (Sekiguchi *et al.*, 2008). We will report herein that that (*R*)-MαNP acid is also a useful auxiliary for the identification of remote asymmetric centers in primary alcohols.

2-Isobutyl-3-phenyl-1-propanol was enantioresolved using (*R*)-(-)-2-methoxy-2-(1-naphthyl)propionic acid and the absolute configuration of the alcohol component of the second fraction from the HPLC separation, the ester C_27_H_32_O_3_ (I) has (Fig. 1) been assigned as *R* from the known configuration of (*R*)-MαNP acid. In the structure of (I) there is a weak intramolecular hydrogen bond linking the naphthyl ring and the methoxy group (C13—H···O1) (Table 1, Fig. 1) which results in the carbon atom of the methyl group lying in the extended plane of the naphthyl ring of the MαNP acid moiety. These structural properties are similar to those of most MαNP acid esters (Kuwahara *et al.*, 2007).

### Experimental

Two diastereomers were obtained from the reaction of (*R*)-(-)-2-methoxy-2-(1-naphthyl)propionic acid with 2-isobutyl-3-phenyl-1-propanol (Kuwahara *et al.*, 2007) and were separated by HPLC, eluting with a mixture of ethyl acetate and hexane (50:1). After removal of most of the solvent, the residual oil was allowed to stand for 6 months, giving single crystals suitable for X-ray diffraction analysis.

### Refinement

In the refinement of the title compound, the H atom positions were calculated geometrically and refined as riding, with C—H bond lengths of 0.95–1.00 Å, and with *U*_iso_(H) values of 1.2*U*_eq_(aromatic C) or 1.5*U*_eq_(methyl C).

### Crystal data

**Table:**

C_27_H_32_O_3_	*F*(000) = 436
*M**_r_* = 404.53	*D*_x_ = 1.185 Mg m^−^^3^
Monoclinic, *P*2_1_	Cu *K*α radiation, λ = 1.54187 Å
Hall symbol: P 2yb	Cell parameters from 21196 reflections
*a* = 9.3380 (1) Å	θ = 3.6–68.3°
*b* = 12.4142 (1) Å	µ = 0.59 mm^−^^1^
*c* = 10.0317 (5) Å	*T* = 105 K
β = 102.8144 (8)°	Prism, colourless
*V* = 1133.95 (6) Å^3^	0.60 × 0.60 × 0.60 mm
*Z* = 2	

### Data collection

**Table:**

Rigaku R-AXIS RAPID CCD diffractometer	4133 independent reflections
Radiation source: rotating anode	4042 reflections with *I* > 2σ(*I*)
graphite	*R*_int_ = 0.026
ω scans	θ_max_ = 68.2°, θ_min_ = 4.5°
Absorption correction: multi-scan (*ABSCOR*; Higashi, 1995)	*h* = −11→11
*T*_min_ = 0.896, *T*_max_ = 1.000	*k* = −14→14
21388 measured reflections	*l* = −12→11

### Refinement

**Table:**

Refinement on *F*^2^	Hydrogen site location: inferred from neighbouring sites
Least-squares matrix: full	H-atom parameters constrained
*R*[*F*^2^ > 2σ(*F*^2^)] = 0.025	*w* = 1/[σ^2^(*F*_o_^2^) + (0.0376*P*)^2^ + 0.1169*P*] where *P* = (*F*_o_^2^ + 2*F*_c_^2^)/3
*wR*(*F*^2^) = 0.068	(Δ/σ)_max_ < 0.001
*S* = 1.06	Δρ_max_ = 0.16 e Å^−^^3^
4133 reflections	Δρ_min_ = −0.11 e Å^−^^3^
276 parameters	Extinction correction: *SHELXL97* (Sheldrick, 2008), Fc^*^=kFc[1+0.001xFc^2^λ^3^/sin(2θ)]^-1/4^
1 restraint	Extinction coefficient: 0.0049 (4)
Primary atom site location: structure-invariant direct methods	Absolute structure: Flack (1983), 1592 Friedel pairs
Secondary atom site location: difference Fourier map	Flack parameter: 0.03 (13)

### Special details

**Table:**

Geometry. All e.s.d.'s (except the e.s.d. in the dihedral angle between two l.s. planes) are estimated using the full covariance matrix. The cell e.s.d.'s are taken into account individually in the estimation of e.s.d.'s in distances, angles and torsion angles; correlations between e.s.d.'s in cell parameters are only used when they are defined by crystal symmetry. An approximate (isotropic) treatment of cell e.s.d.'s is used for estimating e.s.d.'s involving l.s. planes.
Refinement. Refinement of *F*^2^ against ALL reflections. The weighted *R*-factor *wR* and goodness of fit *S* are based on *F*^2^, conventional *R*-factors *R* are based on *F*, with *F* set to zero for negative *F*^2^. The threshold expression of *F*^2^ > σ(*F*^2^) is used only for calculating *R*-factors(gt) *etc*. and is not relevant to the choice of reflections for refinement. *R*-factors based on *F*^2^ are statistically about twice as large as those based on *F*, and *R*- factors based on ALL data will be even larger.

### Fractional atomic coordinates and isotropic or equivalent isotropic displacement parameters (Å^2^)

**Table:**

	*x*	*y*	*z*	*U*_iso_*/*U*_eq_	
C1	0.01084 (12)	0.76296 (10)	0.04854 (12)	0.0259 (2)	
O1	−0.00374 (10)	0.66650 (7)	−0.03129 (9)	0.0328 (2)	
C2	0.01437 (17)	0.67911 (13)	−0.16720 (14)	0.0422 (3)	
H2	−0.0560	0.7325	−0.2148	0.063*	
H2A	−0.0030	0.6100	−0.2152	0.063*	
H2B	0.1146	0.7035	−0.1654	0.063*	
C3	−0.12601 (13)	0.83375 (11)	0.00029 (13)	0.0316 (3)	
H3	−0.1275	0.8614	−0.0915	0.047*	
H3A	−0.1232	0.8943	0.0636	0.047*	
H3B	−0.2145	0.7907	−0.0019	0.047*	
C4	0.00305 (11)	0.72333 (10)	0.19225 (12)	0.0246 (2)	
O2	−0.05044 (9)	0.63985 (7)	0.21644 (9)	0.0328 (2)	
O3	0.05702 (9)	0.79770 (7)	0.28601 (8)	0.02774 (18)	
C5	0.15589 (12)	0.82122 (9)	0.05218 (11)	0.0239 (2)	
C6	0.15720 (13)	0.92718 (10)	0.01434 (12)	0.0278 (3)	
H6	0.0666	0.9633	−0.0185	0.033*	
C7	0.28968 (14)	0.98387 (10)	0.02292 (12)	0.0311 (3)	
H7	0.2874	1.0570	−0.0053	0.037*	
C8	0.42086 (13)	0.93434 (10)	0.07136 (12)	0.0311 (3)	
H8	0.5097	0.9734	0.0782	0.037*	
C9	0.42564 (12)	0.82428 (10)	0.11183 (11)	0.0267 (3)	
C10	0.56161 (13)	0.77226 (11)	0.16393 (12)	0.0319 (3)	
H10	0.6503	0.8121	0.1744	0.038*	
C11	0.56744 (14)	0.66583 (12)	0.19930 (13)	0.0360 (3)	
H11	0.6595	0.6318	0.2330	0.043*	
C12	0.43639 (14)	0.60689 (11)	0.18546 (13)	0.0358 (3)	
H12	0.4404	0.5327	0.2093	0.043*	
C13	0.30273 (13)	0.65541 (10)	0.13778 (12)	0.0295 (3)	
H13	0.2154	0.6144	0.1301	0.035*	
C14	0.29272 (12)	0.76583 (10)	0.09975 (11)	0.0246 (2)	
C15	0.05451 (13)	0.77178 (9)	0.42690 (12)	0.0255 (2)	
H15	0.1298	0.7168	0.4632	0.031*	
H15A	−0.0429	0.7430	0.4323	0.031*	
C16	0.08635 (12)	0.87537 (10)	0.50914 (12)	0.0266 (2)	
H16	0.0802	0.8585	0.6052	0.032*	
C17	−0.02740 (14)	0.96247 (10)	0.45622 (12)	0.0310 (3)	
H17	−0.0271	0.9766	0.3591	0.037*	
H17A	0.0037	1.0297	0.5077	0.037*	
C18	−0.18533 (14)	0.93703 (11)	0.46642 (14)	0.0355 (3)	
H18	−0.2086	0.8617	0.4333	0.043*	
C19	−0.29213 (18)	1.01300 (13)	0.37436 (19)	0.0569 (5)	
H19	−0.3930	0.9941	0.3782	0.085*	
H19A	−0.2803	1.0063	0.2801	0.085*	
H19B	−0.2717	1.0873	0.4057	0.085*	
C20	−0.20296 (16)	0.94390 (14)	0.61282 (16)	0.0480 (4)	
H20	−0.1823	1.0176	0.6467	0.072*	
H20A	−0.1342	0.8941	0.6700	0.072*	
H20B	−0.3038	0.9245	0.6164	0.072*	
C21	0.24294 (14)	0.91710 (10)	0.51362 (13)	0.0313 (3)	
H21	0.2519	0.9334	0.4192	0.038*	
H21A	0.2576	0.9853	0.5661	0.038*	
C22	0.36296 (13)	0.83929 (10)	0.57695 (12)	0.0292 (3)	
C23	0.44401 (13)	0.78389 (11)	0.49766 (13)	0.0330 (3)	
H23	0.4243	0.7959	0.4018	0.040*	
C24	0.55244 (14)	0.71188 (12)	0.55646 (13)	0.0369 (3)	
H24	0.6070	0.6754	0.5008	0.044*	
C25	0.58239 (14)	0.69240 (11)	0.69620 (14)	0.0360 (3)	
H25	0.6571	0.6429	0.7365	0.043*	
C26	0.50175 (13)	0.74623 (12)	0.77605 (13)	0.0366 (3)	
H26	0.5208	0.7333	0.8717	0.044*	
C27	0.39359 (13)	0.81877 (11)	0.71708 (13)	0.0331 (3)	
H27	0.3393	0.8552	0.7730	0.040*	

### Atomic displacement parameters (Å^2^)

**Table:**

	*U*^11^	*U*^22^	*U*^33^	*U*^12^	*U*^13^	*U*^23^
C1	0.0258 (5)	0.0274 (6)	0.0248 (6)	−0.0014 (5)	0.0062 (4)	−0.0059 (5)
O1	0.0376 (5)	0.0321 (5)	0.0302 (4)	−0.0074 (4)	0.0108 (4)	−0.0110 (4)
C2	0.0490 (8)	0.0486 (8)	0.0309 (7)	−0.0055 (6)	0.0133 (6)	−0.0142 (6)
C3	0.0240 (5)	0.0424 (7)	0.0278 (6)	0.0023 (5)	0.0042 (5)	−0.0010 (5)
C4	0.0183 (5)	0.0259 (6)	0.0297 (6)	−0.0001 (4)	0.0056 (4)	−0.0041 (5)
O2	0.0344 (4)	0.0293 (5)	0.0350 (5)	−0.0086 (4)	0.0086 (4)	−0.0035 (4)
O3	0.0350 (4)	0.0253 (4)	0.0245 (4)	−0.0067 (3)	0.0099 (3)	−0.0035 (3)
C5	0.0259 (6)	0.0273 (6)	0.0190 (5)	−0.0003 (4)	0.0065 (4)	−0.0029 (4)
C6	0.0309 (6)	0.0279 (6)	0.0246 (6)	0.0035 (5)	0.0062 (5)	0.0003 (5)
C7	0.0405 (7)	0.0279 (6)	0.0250 (6)	−0.0039 (5)	0.0077 (5)	0.0026 (5)
C8	0.0337 (6)	0.0373 (7)	0.0237 (6)	−0.0115 (5)	0.0090 (5)	−0.0016 (5)
C9	0.0283 (6)	0.0364 (7)	0.0168 (5)	−0.0007 (5)	0.0080 (4)	−0.0012 (5)
C10	0.0244 (5)	0.0497 (8)	0.0228 (6)	−0.0002 (5)	0.0080 (4)	−0.0022 (5)
C11	0.0302 (6)	0.0520 (9)	0.0270 (6)	0.0153 (6)	0.0092 (5)	0.0027 (6)
C12	0.0407 (7)	0.0352 (7)	0.0346 (7)	0.0129 (6)	0.0149 (5)	0.0034 (6)
C13	0.0317 (6)	0.0283 (7)	0.0311 (6)	0.0030 (5)	0.0125 (5)	−0.0002 (5)
C14	0.0268 (6)	0.0284 (6)	0.0201 (5)	0.0017 (4)	0.0082 (4)	−0.0013 (4)
C15	0.0281 (5)	0.0250 (6)	0.0244 (5)	−0.0007 (5)	0.0082 (4)	0.0026 (4)
C16	0.0303 (6)	0.0271 (6)	0.0234 (6)	−0.0007 (5)	0.0079 (4)	0.0003 (5)
C17	0.0425 (7)	0.0249 (6)	0.0249 (6)	0.0024 (5)	0.0061 (5)	−0.0003 (5)
C18	0.0333 (6)	0.0297 (7)	0.0388 (7)	0.0038 (5)	−0.0023 (5)	−0.0079 (6)
C19	0.0455 (9)	0.0390 (9)	0.0714 (11)	0.0092 (7)	−0.0189 (8)	−0.0095 (8)
C20	0.0368 (7)	0.0573 (10)	0.0536 (9)	−0.0032 (7)	0.0178 (6)	−0.0117 (7)
C21	0.0355 (6)	0.0289 (6)	0.0302 (6)	−0.0072 (5)	0.0090 (5)	−0.0029 (5)
C22	0.0274 (6)	0.0320 (6)	0.0276 (6)	−0.0092 (5)	0.0051 (5)	−0.0030 (5)
C23	0.0317 (6)	0.0418 (7)	0.0261 (6)	−0.0055 (6)	0.0077 (5)	−0.0012 (5)
C24	0.0309 (6)	0.0457 (8)	0.0355 (7)	−0.0030 (6)	0.0102 (5)	−0.0037 (6)
C25	0.0267 (6)	0.0427 (8)	0.0361 (7)	−0.0025 (5)	0.0016 (5)	0.0012 (6)
C26	0.0314 (6)	0.0501 (9)	0.0252 (6)	−0.0082 (6)	−0.0001 (5)	−0.0026 (6)
C27	0.0304 (6)	0.0421 (8)	0.0269 (6)	−0.0096 (5)	0.0065 (5)	−0.0104 (5)

### Geometric parameters (Å, °)

**Table:**

C1—O1	1.4303 (14)	C15—C16	1.5211 (16)
C1—C5	1.5288 (15)	C15—H15	0.9900
C1—C3	1.5379 (16)	C15—H15A	0.9900
C1—C4	1.5402 (16)	C16—C17	1.5262 (16)
O1—C2	1.4192 (16)	C16—C21	1.5426 (16)
C2—H2	0.9800	C16—H16	1.0000
C2—H2A	0.9800	C17—C18	1.5337 (18)
C2—H2B	0.9800	C17—H17	0.9900
C3—H3	0.9800	C17—H17A	0.9900
C3—H3A	0.9800	C18—C20	1.516 (2)
C3—H3B	0.9800	C18—C19	1.5258 (19)
C4—O2	1.1984 (14)	C18—H18	1.0000
C4—O3	1.3344 (14)	C19—H19	0.9800
O3—C15	1.4548 (13)	C19—H19A	0.9800
C5—C6	1.3700 (17)	C19—H19B	0.9800
C5—C14	1.4361 (15)	C20—H20	0.9800
C6—C7	1.4092 (17)	C20—H20A	0.9800
C6—H6	0.9500	C20—H20B	0.9800
C7—C8	1.3604 (18)	C21—C22	1.5089 (18)
C7—H7	0.9500	C21—H21	0.9900
C8—C9	1.4233 (18)	C21—H21A	0.9900
C8—H8	0.9500	C22—C27	1.3944 (17)
C9—C10	1.4169 (17)	C22—C23	1.3949 (18)
C9—C14	1.4196 (16)	C23—C24	1.3811 (19)
C10—C11	1.3660 (19)	C23—H23	0.9500
C10—H10	0.9500	C24—C25	1.3885 (19)
C11—C12	1.406 (2)	C24—H24	0.9500
C11—H11	0.9500	C25—C26	1.387 (2)
C12—C13	1.3728 (17)	C25—H25	0.9500
C12—H12	0.9500	C26—C27	1.384 (2)
C13—C14	1.4204 (17)	C26—H26	0.9500
C13—H13	0.9500	C27—H27	0.9500
			
O1—C1—C5	112.53 (9)	C16—C15—H15A	110.3
O1—C1—C3	109.45 (9)	H15—C15—H15A	108.5
C5—C1—C3	114.03 (10)	C15—C16—C17	111.91 (9)
O1—C1—C4	103.79 (9)	C15—C16—C21	111.70 (9)
C5—C1—C4	110.75 (9)	C17—C16—C21	110.75 (10)
C3—C1—C4	105.59 (9)	C15—C16—H16	107.4
C2—O1—C1	115.39 (10)	C17—C16—H16	107.4
O1—C2—H2	109.5	C21—C16—H16	107.4
O1—C2—H2A	109.5	C16—C17—C18	115.86 (10)
H2—C2—H2A	109.5	C16—C17—H17	108.3
O1—C2—H2B	109.5	C18—C17—H17	108.3
H2—C2—H2B	109.5	C16—C17—H17A	108.3
H2A—C2—H2B	109.5	C18—C17—H17A	108.3
C1—C3—H3	109.5	H17—C17—H17A	107.4
C1—C3—H3A	109.5	C20—C18—C19	110.78 (13)
H3—C3—H3A	109.5	C20—C18—C17	111.45 (11)
C1—C3—H3B	109.5	C19—C18—C17	109.96 (12)
H3—C3—H3B	109.5	C20—C18—H18	108.2
H3A—C3—H3B	109.5	C19—C18—H18	108.2
O2—C4—O3	124.39 (11)	C17—C18—H18	108.2
O2—C4—C1	125.03 (10)	C18—C19—H19	109.5
O3—C4—C1	110.48 (9)	C18—C19—H19A	109.5
C4—O3—C15	116.54 (9)	H19—C19—H19A	109.5
C6—C5—C14	119.33 (10)	C18—C19—H19B	109.5
C6—C5—C1	120.66 (10)	H19—C19—H19B	109.5
C14—C5—C1	119.98 (10)	H19A—C19—H19B	109.5
C5—C6—C7	121.62 (11)	C18—C20—H20	109.5
C5—C6—H6	119.2	C18—C20—H20A	109.5
C7—C6—H6	119.2	H20—C20—H20A	109.5
C8—C7—C6	120.32 (11)	C18—C20—H20B	109.5
C8—C7—H7	119.8	H20—C20—H20B	109.5
C6—C7—H7	119.8	H20A—C20—H20B	109.5
C7—C8—C9	120.31 (11)	C22—C21—C16	114.08 (10)
C7—C8—H8	119.8	C22—C21—H21	108.7
C9—C8—H8	119.8	C16—C21—H21	108.7
C10—C9—C14	119.58 (11)	C22—C21—H21A	108.7
C10—C9—C8	120.77 (11)	C16—C21—H21A	108.7
C14—C9—C8	119.65 (11)	H21—C21—H21A	107.6
C11—C10—C9	121.16 (12)	C27—C22—C23	117.96 (12)
C11—C10—H10	119.4	C27—C22—C21	120.57 (11)
C9—C10—H10	119.4	C23—C22—C21	121.45 (11)
C10—C11—C12	119.61 (12)	C24—C23—C22	120.98 (12)
C10—C11—H11	120.2	C24—C23—H23	119.5
C12—C11—H11	120.2	C22—C23—H23	119.5
C13—C12—C11	120.68 (13)	C23—C24—C25	120.55 (12)
C13—C12—H12	119.7	C23—C24—H24	119.7
C11—C12—H12	119.7	C25—C24—H24	119.7
C12—C13—C14	121.17 (12)	C26—C25—C24	119.06 (13)
C12—C13—H13	119.4	C26—C25—H25	120.5
C14—C13—H13	119.4	C24—C25—H25	120.5
C9—C14—C13	117.78 (10)	C27—C26—C25	120.33 (12)
C9—C14—C5	118.70 (10)	C27—C26—H26	119.8
C13—C14—C5	123.50 (10)	C25—C26—H26	119.8
O3—C15—C16	107.26 (9)	C26—C27—C22	121.11 (12)
O3—C15—H15	110.3	C26—C27—H27	119.4
C16—C15—H15	110.3	C22—C27—H27	119.4
O3—C15—H15A	110.3		
			
C5—C1—O1—C2	−54.27 (13)	C8—C9—C14—C13	178.30 (10)
C3—C1—O1—C2	73.61 (13)	C10—C9—C14—C5	177.39 (10)
C4—C1—O1—C2	−174.05 (10)	C8—C9—C14—C5	−2.68 (16)
O1—C1—C4—O2	−21.54 (14)	C12—C13—C14—C9	0.39 (16)
C5—C1—C4—O2	−142.53 (11)	C12—C13—C14—C5	−178.57 (11)
C3—C1—C4—O2	93.57 (13)	C6—C5—C14—C9	2.77 (16)
O1—C1—C4—O3	161.89 (8)	C1—C5—C14—C9	−175.26 (9)
C5—C1—C4—O3	40.90 (12)	C6—C5—C14—C13	−178.28 (11)
C3—C1—C4—O3	−83.00 (11)	C1—C5—C14—C13	3.69 (17)
O2—C4—O3—C15	2.47 (16)	C4—O3—C15—C16	−166.99 (9)
C1—C4—O3—C15	179.06 (9)	O3—C15—C16—C17	59.82 (12)
O1—C1—C5—C6	125.29 (11)	O3—C15—C16—C21	−64.99 (12)
C3—C1—C5—C6	−0.14 (15)	C15—C16—C17—C18	63.25 (13)
C4—C1—C5—C6	−119.05 (11)	C21—C16—C17—C18	−171.41 (10)
O1—C1—C5—C14	−56.71 (13)	C16—C17—C18—C20	73.35 (14)
C3—C1—C5—C14	177.87 (10)	C16—C17—C18—C19	−163.40 (11)
C4—C1—C5—C14	58.95 (13)	C15—C16—C21—C22	−59.85 (13)
C14—C5—C6—C7	−1.01 (17)	C17—C16—C21—C22	174.69 (10)
C1—C5—C6—C7	177.01 (10)	C16—C21—C22—C27	−70.82 (14)
C5—C6—C7—C8	−0.91 (18)	C16—C21—C22—C23	107.61 (13)
C6—C7—C8—C9	0.99 (17)	C27—C22—C23—C24	−0.76 (18)
C7—C8—C9—C10	−179.25 (11)	C21—C22—C23—C24	−179.22 (12)
C7—C8—C9—C14	0.82 (16)	C22—C23—C24—C25	0.5 (2)
C14—C9—C10—C11	1.84 (17)	C23—C24—C25—C26	0.1 (2)
C8—C9—C10—C11	−178.08 (11)	C24—C25—C26—C27	−0.4 (2)
C9—C10—C11—C12	−0.77 (18)	C25—C26—C27—C22	0.12 (19)
C10—C11—C12—C13	−0.50 (19)	C23—C22—C27—C26	0.44 (18)
C11—C12—C13—C14	0.68 (18)	C21—C22—C27—C26	178.92 (11)
C10—C9—C14—C13	−1.62 (16)		

### Hydrogen-bond geometry (Å, °)

**Table:**

*D*—H···*A*	*D*—H	H···*A*	*D*···*A*	*D*—H···*A*
C13—H13···O1	0.95	2.40	2.9887 (15)	120
